# Changes in First-Line cART Regimens and Short-Term Clinical Outcome between 1996 and 2010 in The Netherlands

**DOI:** 10.1371/journal.pone.0076071

**Published:** 2013-09-30

**Authors:** Mikaela Smit, Colette Smit, Suzanne Geerlings, Luuk Gras, Kees Brinkman, Timothy B. Hallett, Frank de Wolf, on behalf of the Athena Observational Cohort

**Affiliations:** 1 Department of Infectious Disease Epidemiology, Imperial College, Faculty of Medicine, London, United Kingdom; 2 HIV Monitoring Foundation, Amsterdam, The Netherlands; 3 Division of Infectious, Diseases Amsterdam Medical Centre, Amsterdam, The Netherlands; 4 Department of Internal Medicine, Onze Lieve Vrouwe Gasthuis, Amsterdam, The Netherlands; University of Pittsburgh, United States of America

## Abstract

**Objectives:**

Document progress in HIV-treatment in the Netherlands since 1996 by reviewing changing patterns of cART use and relating those to trends in patients' short-term clinical outcomes between 1996 and 2010.

**Design and Methods:**

1996–2010 data from 10,278 patients in the Dutch ATHENA national observational cohort were analysed. The annual number of patients starting a type of regimen was quantified. Trends in the following outcomes were described: i) recovery of 150 CD4 cells/mm^3^ within 12 months of starting cART; ii) achieving viral load (VL) suppression ≤1,000 copies/ml within 12 months of starting cART; iii) switching from first-line to second-line regimen within three years of starting treatment; and iv) all-cause mortality rate per 100 person-years within three years of starting treatment.

**Results:**

Between 1996 and 2010, first-line regimens changed from lamivudine/zidovudine-based or lamivudine/stavudine-based regimens with unboosted-PIs to tenofovir with either emtricitabine or lamivudine with NNRTIs. Mortality rates did not change significantly over time. VL suppression and CD4 recovery improved over time, and the incidence of switching due to virological failure and toxicity more than halved between 1996 and 2010. These effects appear to be related to the use of new regimens rather than improvements in clinical care.

**Conclusion:**

The use of first-line cART in the Netherlands closely follows changes in guidelines, to the benefit of patients. While there was no significant improvement in mortality, newer drugs with better tolerability and simpler dosing resulted in improved immunological and virological recovery and reduced incidences of switching due to toxicity and virological failure.

## Introduction

Combination antiretroviral therapy (cART) was first introduced more than 15 years ago. Since 1996, over twenty new antiretroviral drugs have been licenced for the treatment of HIV-infection [1]. A number of clinical trials and studies have compared specific antiretroviral drugs or regimen types with respect to selected clinical outcomes [2]–[12]. However, it remains unclear how the improved efficacy of new antiretroviral reported in trials has translated to population-level effectiveness in general clinical care.

A non-selective database that collects data from all HIV-infected patients in clinical care in the Netherlands provides a unique opportunity to record the progress of cART since 1996 across a variety of clinical and non-clinical markers. We aim to use this dataset to document progress in HIV-treatment by: i) reviewing the changing patterns of first-line cART regimens between 1996 and 2010 in the Netherlands; ii) to describe time trends in a variety of short-term clinical and non-clinical markers; and iii) to relate new regimens to trends in patients' short-term clinical outcomes as a measure of population-level effectiveness.

## Methods

### Data

ATHENA is a national observational cohort that includes all HIV-patients followed in 25 designated HIV treatment centres in the Netherlands since 1996. The design of this cohort has been described previously [13]. Clinical, biological and immunological data on HIV-infected patients are collected upon entry and at each follow-up visit. Anonymised patient data are available on request and for scientific research purposes only (Ref: www.hiv-monitoring.nl).

Patients from the ATHENA cohort were included in this analysis if they were aged 18 or over, infected with HIV-1, and diagnosed with HIV from 1^st^ January 1996. Patients were antiretroviral drug-naïve prior to entering the study and started cART during follow-up. Women known to have been pregnant during follow-up were excluded. Data was analyzed up to and including 31^st^ December 2010. The number of patients in the analysis was 10 278 patients.

Combination therapy was defined as regimens containing three or more antiretroviral drugs. Regimen types were classified by nucleoside reverse transcriptase inhibitor (NRTI) backbone: i) lamivudine (3TC) and stavudine (d4T); ii) 3TC and zidovudine (AZT); and iii) tenofovir (TDF) with 3TC or emtricitabine (FTC). The analysis was further stratified into regimens combined with either: i) non-nucleoside reverse transcriptase inhibitors (NNRTI); ii) non-boosted protease inhibitors (PI); or iii) (ritonavir)-boosted-PIs. Regimens other than those described above were classified as ‘other’ in further analysis. The choice of regimen classification reflects the main regimen types used between 1996 and 2010 in the Netherlands.

Clinical outcome was measured using: i) hazard rate of recovering 150 CD4 cells/mm^3^ within 12 months of starting cART; ii) hazard rate of achieving viral load (VL) suppression ≤1,000 copies/ml within 12 months of starting cART; iii) the incidence of switching from first-line to second-line regimen within three years of starting treatment; and iv) all-cause mortality rate per 100 person-years within three years of starting treatment. A threshold of 1,000 copies/ml for VL suppression was chosen to allow for comparison across the years, due to the changing detection threshold for VL suppression, from 1,000 copies/ml in 1996 to 20 copies/ml in 2010.

A switch was defined as a change in regimen that included at least one new antiretroviral drug. Reasons for switching were classified as: i) toxicity, ii) simplification/new medication becoming available, iii) virological failure, and iv) other reasons [14]. ‘Toxicity’ is defined as the need to change regimen due to experience of side effects. ‘Simplification/new medication becomes available’ is defined as patients switching regimen for a simpler cART regimen, for example a once-daily regimen, or because a new cART regimen has become available. ‘Virological failure’ refers to a switch due to poor virological response, including resistance. ‘Other reasons’ included pharmacological reasons, caution, ‘other’ and ‘unknown’ reasons as classified by the ATHENA cohort. ‘Pharmacologic reason’ refers to an interaction with co-medication/other drug and ‘caution’ refers to the situation where patients are starting an additional treatment, for example chemotherapy, and the physician decides to stop or change a regimen as the combination of side effects may be too heavy. Apart from toxicity, simplification/new medication becoming available, virological failure, and other reasons, there were other drug-related reasons for switching treatment (compliance (0.66%), and contra-indication (0.04%)), which were excluded as they accounted for less than 1% of switches. Date and reasons for switching are recorded by physicians at follow-up visit when a patient is prescribed a change in cART regimen.

### Statistical analysis

Time-dependent Cox proportional hazard model and Kaplan-Meier curves were used as time-to-event analysis. Cox regression analyses were carried out on all four clinical outcomes defined above. When analysing switching, Cox analysis was limited to virological failure and toxicity. Follow-up time was defined as months from the start of treatment until the date of end of follow-up time, closure of the database (31^st^ December 2010), or the outcome of interest, whichever occurred first. Follow-up was divided into three time periods, 1996–2000, 2001–2005, and 2006–2010, early cART, medium cART and late cART period. Mortality was treated as a censored event in analysis where mortality was not the outcome of interest. Wald statistics were used to determine significance of this analysis using a significance level of 0.05 throughout.

Cox Proportional Hazard analysis were adjusted by sex, age (divided into 5-year categories,18–22, 23–27 years, etc., the last category being 78–82 years), CD4 and VL at the start of treatment, route of HIV transmission (men-who-have-sex-with-men (MSM), heterosexual, injecting drug use (IDU), and vertical infection, blood infusion, or unknown), and region of origin. Categories of regions of origin were Netherlands, Europe (excluding the Netherlands), Sub-Saharan Africa, and other. CD4 counts categories were <200, 201–350, 351–500, and >501 cells/mm^3^, and VL categories were <100,000, 100,000–1,000,000, and >1,000,000 copies/ml. Analysis of mortality was further controlled for smoking and cumulative number of AIDS-defining events, as a proxy for lifestyle and advanced disease. Dutch-born MSM with a CD4 between 350 and 500 cells/mm^3^ and RNA below 100,000 copies/ml when starting cART were used as the reference categories in the Cox model. The reference regimen type was TDF/3TC or TDF/FTC with a NNRTI. These references were chosen, as this constitutes the majority of patients in the cohort, with the CD4 range of current guidelines for treatment initiation [15]. All the data-analysis was carried out in SAS v.9.3 (SAS Institute, Cary, North Carolina, USA).

## Results

### Population Description

Demographic characteristics of the patients are summarized in Table 1. Overall, of the 10,278 patients 84% were male, 59% were MSM, and 58% were Dutch-born. 44% of patients had spent a cumulative time five to ten years on cART, and the mean age was 40 at the start of treatment. 391 patients died within three years of starting treatment.

**Table 1:** Demographic characteristics of the 10,278 patients in the study population by calendar time of treatment initiation.

	1996–2000		2001–2005		2006–2010		Total	
	(N = 1,997, 19%)		(N = 3,190, 31%)		(N = 5,091, 50%)		(N = 10,278)	
**Sex**								
Men	1,721	86	2,543	80	4,322	85	8,586	84
Women	276	14	647	20	769	15	1,692	16
**Mean age (years) at treatment initiation**	39 (IQR, 32–44)		39 (IQR, 33–46)		41 (IQR, 34–48)		40 (IQR, 33–47)	
**Transmission**								
MSM	1,186	59	1,575	49	3,279	64	6,040	59
Heterosexual	605	30	1,247	39	1,448	28	3,300	32
IDU	70	4	75	2	63	1	208	2
Other/unknown	136	7	293	9	301	6	730	7
**Region of origin**								
The Netherlands	1,188	59	1,643	52	3,136	62	5,947	58
Sub-Saharan Africa	264	13	717	22	647	13	1,628	16
Europe	188	9	230	7	437	9	855	8
Other	357	18	600	19	871	17	1,828	18
**Cumulative years on treatment**								
**<1**	29	1	76	2	513	10	618	6
**1**–**2**	41	2	51	2	1,495	29	1,587	15
**3**–**4**	29	1	43	1	1,633	32	1,705	17
**5**–**10**	391	20	2,774	87	1,331	26	4,497	44
**>10**	1,506	75	246	8	119	2	1,871	18
**Number of deaths within three years of treatment initiation**	75	4	154	5	162	3	391	4

### cART regimens between 1996 and 2010

**Figure 1:**
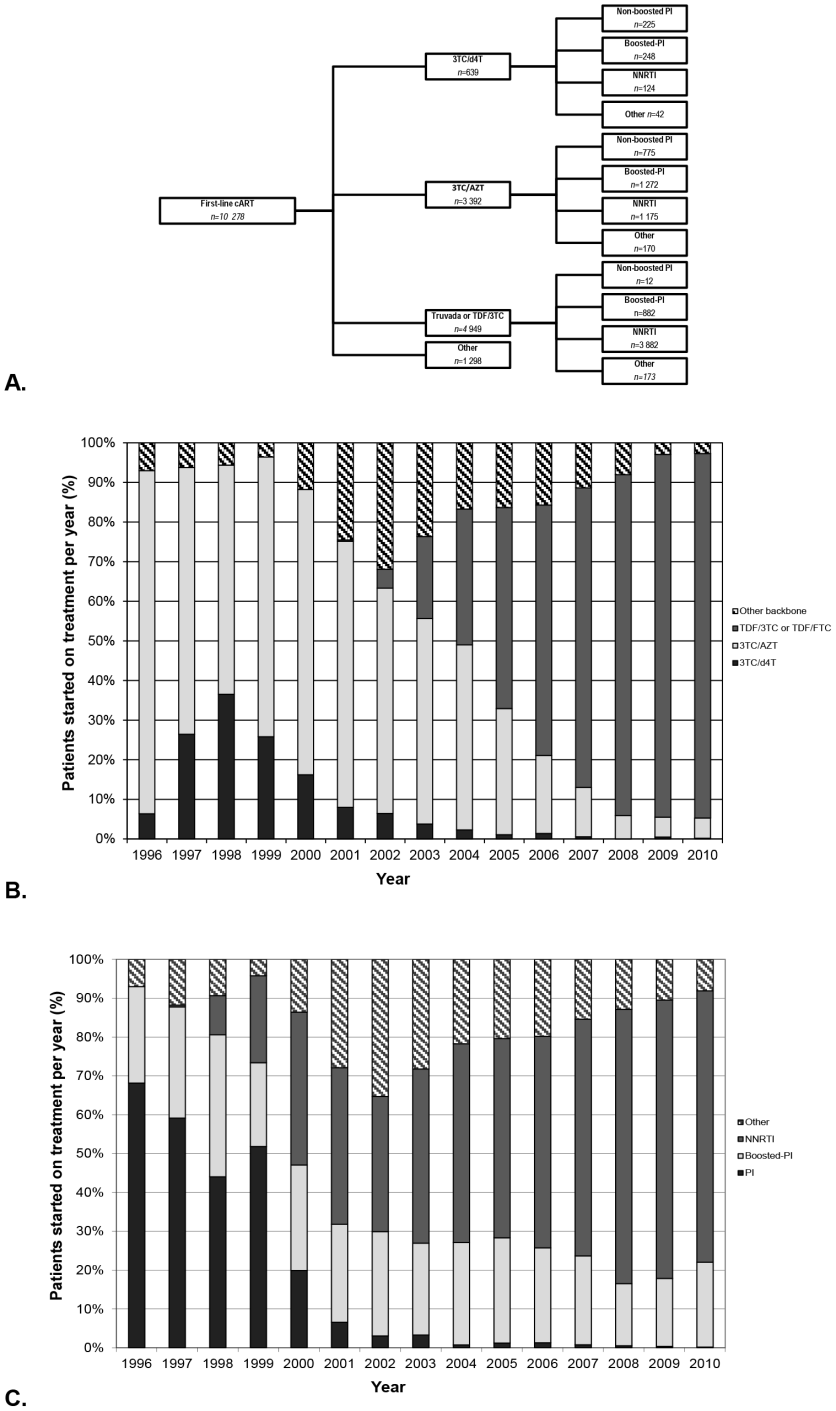
First-line cART regimens. A. First-line cART regimens prescribed in the Netherlands between 1996 and 2010. Relative distribution of patients starting first-line regimens per year by **B**. NRTI backbone and **C**. third cART component.

The cART regimen types used between 1996 and 2010 are presented in Figure 1A. A backbone of 3TC/d4T was most commonly combined with boosted-PIs (n = 248, 39%) or PIs (n = 225, 35%). 3TC/AZT was most frequently combined with boosted-PIs (n = 1,272, 38%) or a NNRTI (n = 1,175, 35%), while TDF/3TC or TDF/FTC was mostly combined with NNRTIs (n = 3,882, 78%). 16.4% of patients started on regimen types with other backbones or third components.

Between 1996 and 2010, the number of patients starting treatment increased. In 1996, 156 patients started on therapy per year, increasing to around 1,000 patients per year after 2007. From 1996 to 2004 the majority of patients started on a backbone of 3TC/AZT (Figure 1B and C). Its use declined steadily after 2001, accounting for less than 10% of NRTI backbone from 2008. The use of 3TC/d4T ranged from 7% to 36% between 1996 and 2002, and accounted for less than 10% of new prescriptions from 2003. Other backbones or other third components were most commonly prescribed between 2001 and 2004, accounting for up to 35.3% or first-line regimes. Since the introduction of TDF in Europe in 2001, its use as a first-line regimen NRTI backbone increased steadily. After 2004, TDF/3TC or TDF/FTC-based regimens constituted for the majority of NRTI-backbones for first-line in the Netherlands accounting for over 85% of first-line backbones from 2007. The most frequently used third component from 1996 to 2000 were PIs (45–81% non-boosted PIs, 9–48% boosted-PIs), and from 2000–2010 were NNRTIs (45–81%) (Figure 1C).

### Clinical outcomes between 1996 and 2010

#### Mortality

**Figure 2:**
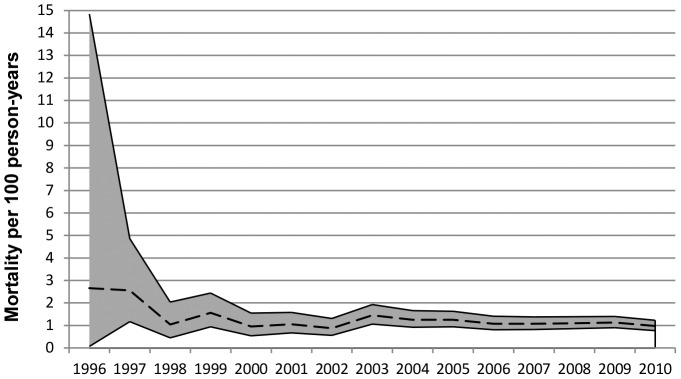
Mortality rate per 100 person-years. The black dotted line is the mortality rate; the grey shaded area represents the 95% confidence-intervals.

The Kaplan-Meier estimate of the percentage of deaths at three years was 6.4% (95% CI 5.9–6.9). The short-term all-cause mortality rate of patients on cART between 1996 and 2010 is presented in Figure 2. Hazards for mortality did not differ significantly between 1996 and 2010 (Table S1) (p-value = 0.44 for 1996–2000 and p-value = 0.16 for 2001–2005 compared to 2006–2010).

All-cause mortality was significantly increased in older patients (HR = 1.27 per 5-year increase in age, 95% CI 1.21–1.33, p-value<.0001), in patients who had acquired HIV via injecting drug use compared to MSM (HR = 2.90, 95%CI 1.81–4.64, p-value<0.0001), in smokers (HR = 1.70, 95% CI 1.11–2.60, p-value = 0.01), and with increasing cumulative AIDS-defining events (p-value<0.0001). Patients on regimens of TDF/3TC or TDF/FTC with boosted-PIs may experience increased risk of mortality compared to patients on TDF/3TC or TDF/FTC with NNRTIs (HR = 1.49, 95% CI 1.03–2.15, p-value = 0.04).

#### CD4 recovery

**Figure 3:**
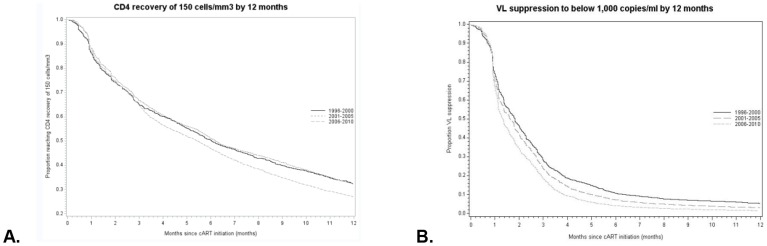
Rates of CD4 recovery and VL suppression. Unadjusted Kaplan-Meier curves for A) Rates of CD4 recovery of 150 cells/mm^3^ by 12 months and B) Rates of VL suppression to below 1,000 copies/ml by 12 months.

Between 1996 and 2010 70.4% (95% CI 69.5–71.3) patients reached a CD4 increase of 150 cells/mm^3^ within 12 months of treatment initiation. The CD4 recovery rate of 150 cells/mm^3^ following treatment initiation was significantly higher in 2006–2010 compared to 2001–2005 (HR = 0.94, 95% CI 0.88–0.99, p-value = 0.03) (Figure 3A, Table S2). The difference between periods was not significant when models were adjusted for cART regimen (p-value = 0.46).

CD4 recovery of 150 cells/mm^3^ was improved in women (HR = 1.26, 95% CI 1.14–1.38, p-value<0.0001 for women), worse in older patients (HR = 0.97 per 5-year increase in age, 95% CI 0.96–0.99, p-value = 0.0001), patients from Sub-Saharan Africa compared to Dutch-born (HR = 0.71, 95% CI 0.64–0.78, p-value<0.0001), and patients who had acquired HIV through heterosexual contact or injecting drug use compared to MSM (HR = 0.80, 95%CI 0.74–0.87, p-value<0.0001 and HR = 0.51, 95% CI 0.39–0.67, p-value<0.0001, respectively). CD4 recovery was also worse in patients who had a CD4 count below 200 cell/mm^3^ or above 500 cells/mm^3^ and VL below 100 000 copies/ml at the start of treatment (HR = 0.84, 95%CI 0.77–0.92, p-value = 0.0003 and HR = 0.76, 95%CI 0.65–0.89, p-value = 0.0004, respectively). Patients on regimens of 3TC/d4T or TDF/3TC or TDF/FTC with boosted-PIs may experience improved rates of immunological recovery, compared to patients on TDF/3TC or TDF/FTC with NNRTIs (HR = 1.30, 95%CI 1.08–1.57, p-value = 0.01 and HR = 1.18, 95%CI 1.07–1.29, p-value = 0.0006).

#### Viral Load suppression

The Kaplan-Meier estimate of the percentage of patients achieving VL suppression <1,000 copies/ml by 12 months was 94.7% (95% CI 94.2–95.1). VL suppression following the start of cART was improved in recent years (HR = 0.79, 95% CI 0.75–0.84, p-value<0.0001 for 1996–2000 and HR = 0.90, 95% CI 0.86–0.95, p-value<0.0001 for 2001–2005) (Figure 3B and Table S3). The difference between periods became non-significant when models were adjusted for cART regimen type (p-value = 0.07 and p-value = 0.12).

In adjusted analysis, VL suppression was worse in patients non-Dutch, European patients (HR = 0.90, 95% CI 0.82–0.98, p-value = 0.02), for those who had acquired HIV either via heterosexual contact or injecting drug use (HR = 0.88, 95% CI 0.82–0.95, p-value = 0.0004 and HR = 0.78, 95% CI 0.64–0.94, p-value = 0.01), and in patients with a VL above 100,000 copies/ml at the start of treatment (p-value<0.0001). Regimens of 3TC/AZT with non-boosted or boosted-PIs, and TDF/3TC or TDF/FTC with boosted-PIs may be associated with worse rates of VL suppression (HR = 0.71, 95%CI 0.63–0.81, p-value<0.0001, HR = 0.82, 95%CI 0.75–0.89, p-value<0.0001, and HR = 0.87, 95% CI 0.81–0.95, p-value = 0.001).

#### Incidence of switching

**Figure 4:**
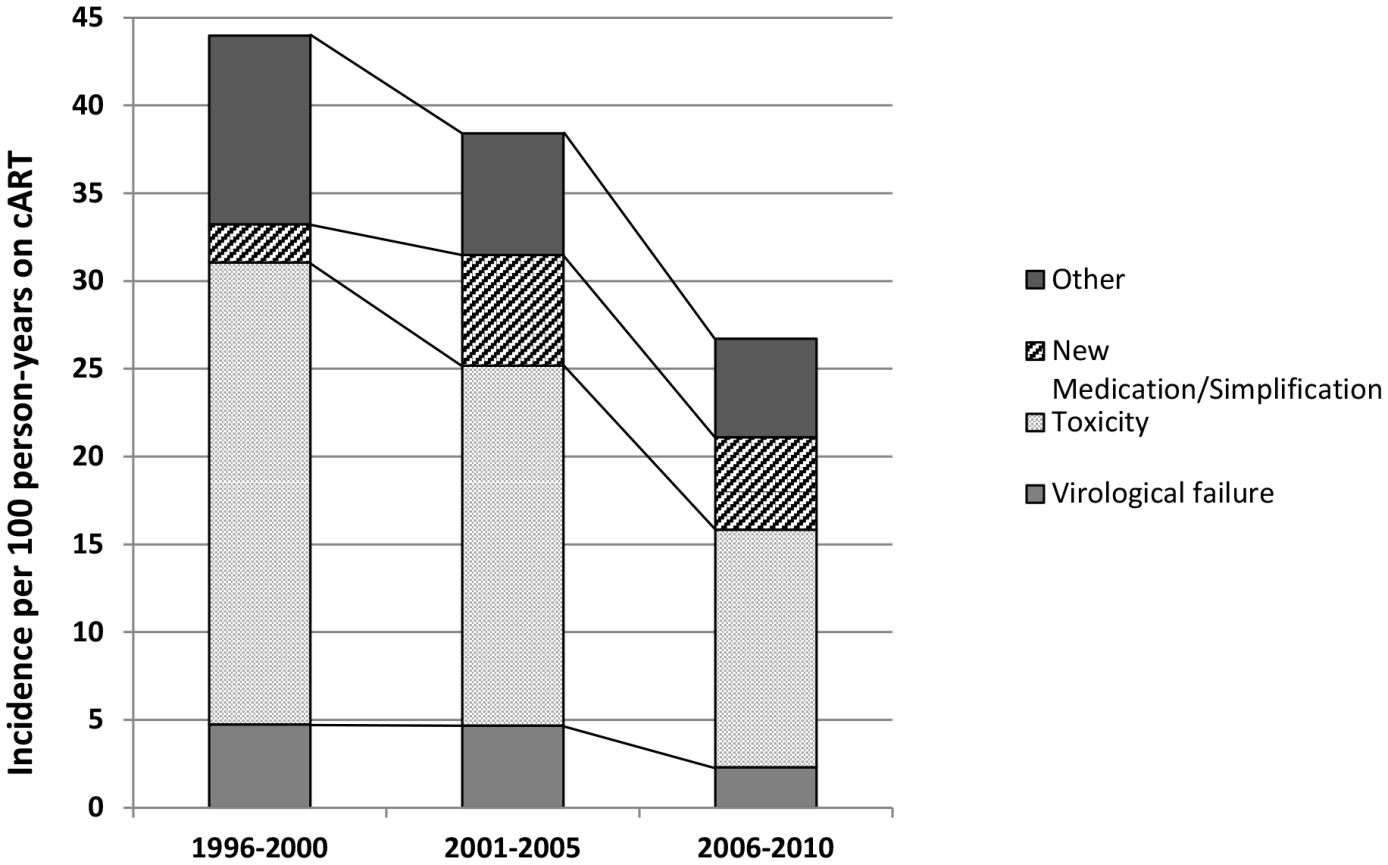
Rate of switching per 100 person-years. Calendar time refers to time of switching.

During follow-up, 4,481 patients (44%) switched to second-line. The short-term incidence of switching, due to virological failure or toxicity, from first-line regimens has declined between 1996 and 2010 (Figure 4), suggesting that the duration patients spend on a cART-line before switching regimens has improved considerably over time. The incidence of switching due to virological failure, toxicity, and ‘other’ reasons between 2006 and 2010 was less than half that of 1996 to 2000. The incidence of switching due to virological failure decreased from 4.7 events (95% CI 3.8–5.8) in 1996–2000 to 2.3 events per 100 person-years (95% CI 2.0–2.7) in 2006–2010. The incidence of toxicity-related switching decreased from 26.3 events (95% CI 24.1–28.7) in 1996–2000 to 13.5 events per 100 person-years (95% CI 12.7–14.4) in 2006–2010. In contrast, the incidence of switching due to simplification/new medication becoming available increased between 1996 and 2010 from 2.2 events per 100 person-years (95% CI 1.6–2.95) in 1996-2000 to 5.26 events per 100 person-years (95% CI 4.75–5.81) in 2006–2010.

In models adjusted for cART regimen type, differences between time periods were not significant (Table 2 and Table 3). The only exception was the increased incidence of switching due to virological failure amongst patient who started first-line in 2001–2005 compared to 2006–2010, which could partly, but not fully, be attributed to the newer regimens (Table 3).

**Table 2:** Adjusted hazard ratio (95% Confidence intervals) of switching from first-line to second-line due to toxicity.

Variables	Model 1: Calendar time		Model 2: Calendar time and regimen type	
	Hazard Ratio (95% CI)	P-value	Hazard Ratio (95% CI)	P-value
**Calendar period**				
1996–2000	1.53 (1.37–1.71)	<.0001	1.02 (0.84–1.24)	0.83
2001–2005	1.40 (1.27–1.54)	<.0001	1.00 (0.86–1.16)	0.99
2006–2010	[Reference]		[Reference]	
**Demographic**				
**Age**				
5-year increased from 18 years old	1.04 (1.02–1.06)	0.0007	1.04 (1.02–1.07)	0.001
**Sex**				
Male	[Reference]		[Reference]	
Female	1.36 (1.18–1.56)	<.0001	1.39 (1.19–1.62)	<.0001
**Region of Origin**				
Netherlands	[Reference]		[Reference]	
European	0.86 (0.73–1.02)	0.09	0.87 (0.72–1.04)	0.12
Sub-Saharan Africa	1.04 (0.90–1.21)	0.57	1.02 (0.86–1.19)	0.85
Other	1.01 (0.90–1.13)	0.90	0.99 (0.87–1.12)	0.82
**Route of transmission**				
Heterosexual	1.21 (1.07–1.37)	0.003	0.80 (0.70–0.92)	0.002
**MSM**	[Reference]		[Reference]	
Injecting Drug Use	0.93 (0.64–1.34)	0.69	0.75 (0.49–1.13)	0.17
Other	1.03 (0.86–1.23)	0.78	0.87 (0.72–1.05)	0.14
**Clinical**				
**CD4 cell count at start of cART**				
CD4<200	0.99 (0.86–1.14)	0.90	0.96 (0.82–1.12)	0.61
CD4 201–350	0.93 (0.81–1.07)	0.30	0.96 (0.82–1.12)	0.57
CD4 351–500	[Reference]		[Reference]	
CD4>501	1.44 (1.19–1.75)	0.0002	1.24 (0.99–1.56)	0.06
**RNA at start of cART**				
RNA<100 000	[Reference]		[Reference]	
RNA 100 000–1 000 000	1.05 (0.96–1.15)	0.32	0.97 (0.87–1.07)	0.50
RNA>1 000 000	1.08 (0.88–1.32)	0.47	0.88 (0.69–1.12)	0.29
**cART Type**				
3TC/d4T+PI			2.16 (1.63–2.86)	<.0001
3TC/d4T+Boosted-PI			3.34 (2.59–4.31)	<.0001
3TC/d4T+NNRTI			2.21 (1.50–3.26)	<.0001
3TC/AZT+PI			1.38 (1.08–1.75)	0.01
3TC/AZT+Boosted-PI			2.31 (1.97–2.70)	<.0001
3TC/AZT+NNRTI			1.55 (1.30–1.86)	<.0001
TDF/FTC or TDF/3TC+Boosted-PI			1.16 (0.97–1.39)	0.10
TDF/FTC or TDF/3TC+NNRTI			[Reference]	

**Table 3:** Adjusted hazard ratio (95% Confidence intervals) of switching from first-line to second-line due to virological failure.

Variables	Model 1: Calendar time		Model 2: Calendar time and regimen type	
	Hazard Ratio (95% CI)	P-value	Hazard Ratio (95% CI)	P-value
**Calendar period**				
1996–2000	1.69 (1.30–2.23)	0.0002	0.83 (0.50–1.35)	0.45
2001–2005	1.81 (1.46–2.25)	<.0001	1.86 (1.31–2.62)	0.001
2006–2010	[Reference]		[Reference]	
**Demographic**				
**Age**				
5-year increased from 18 years old	0.95 (0.91–1.01)	0.07	0.98 (0.93–1.04)	0.47
**Sex**				
Male	[Reference]		[Reference]	
Female	0.91 (0.67–1.23)	0.53	0.74 (0.52–1.04)	0.08
**Region of Origin**				
Netherlands	[Reference]		[Reference]	
European	0.91 (0.61–1.35)	0.63	0.94 (0.61–1.45)	0.79
Sub-Saharan Africa	1.28 (0.94–1.75)	0.11	1.48 (1.06–2.08)	0.02
Other	1.03 (0.78–1.35)	0.85	1.08 (0.80–1.45)	0.61
**Route of transmission**				
Heterosexual	0.90 (0.69–1.17)	0.42	1.10 (0.82–1.46)	0.54
MSM	[Reference]		[Reference]	
Injecting Drug Use	0.85 (0.37–1.96)	0.71	0.82 (0.30–2.23)	0.69
Other	0.84 (0.57–1.24)	0.37	0.77 (0.49–1.21)	0.25
**Clinical**				
**CD4 cell count at start of cART**				
CD4<200	2.92 (1.83–4.67)	<.0001	3.16 (1.83–5.46)	<.0001
CD4 201–350	1.50 (0.92–2.44)	0.11	1.64 (0.93–2.89)	0.09
CD4 351–500	[Reference]		[Reference]	
CD4 >501	1.36 (0.66–2.81)	0.40	1.20 (0.50–2.86)	0.69
**RNA at start of cART**				
RNA<100 000	[Reference]		[Reference]	
RNA 100 000–1 000 000	1.60 (1.27–2.01)	<.0001	1.87 (1.44–2.43)	<.0001
RNA >1 000 000	2.46 (1.68–3.60)	<.0001	3.3 (2.16–5.13)	<.0001
**cART Type**				
3TC/d4T+PI			3.05 (1.79–5.19)	<.0001
3TC/d4T+Boosted-PI			0.87 (0.40–1.90)	0.72
3TC/d4T+NNRTI			1.03 (0.43–2.44)	0.95
3TC/AZT+PI			2.65 (1.65–4.24)	<.0001
3TC/AZT+Boosted-PI			0.60 (0.40–0.92)	0.02
3TC/AZT+NNRTI			0.69 (0.45–1.04)	0.08
TDF/FTC or TDF/3TC+Boosted-PI			0.51 (0.30–0.87)	0.01
TDF/FTC or TDF/3TC+NNRTI			[Reference]	

Older patients and women had an increased risk of toxicity-driven switch, while patients who acquired HIV via heterosexual sex had a reduced risk of toxicity-driven switch compared to MSM. Patients on backbones of 3TC with either d4T or AZT had an increased risk of toxicity-driven switch compared to patients on TDF/3TC or TDF/FTC with boosted-PIs. The incidence of switching due to virological failure was higher in patients from Sub-Saharan Africa, and with a CD4 count below 200 cells/mm^3^ and VL above 100,000 copies/ml at the start of treatment. Patients on regimens of 3TC/d4T or 3TC/AZT with unboosted-PIs had an increased risk of virological failure, while patients on 3TC/AZT or TDF/3TC or TDF/FTC with boosted-PIs had a decreased risk of virological failure.

**Figure 5:**
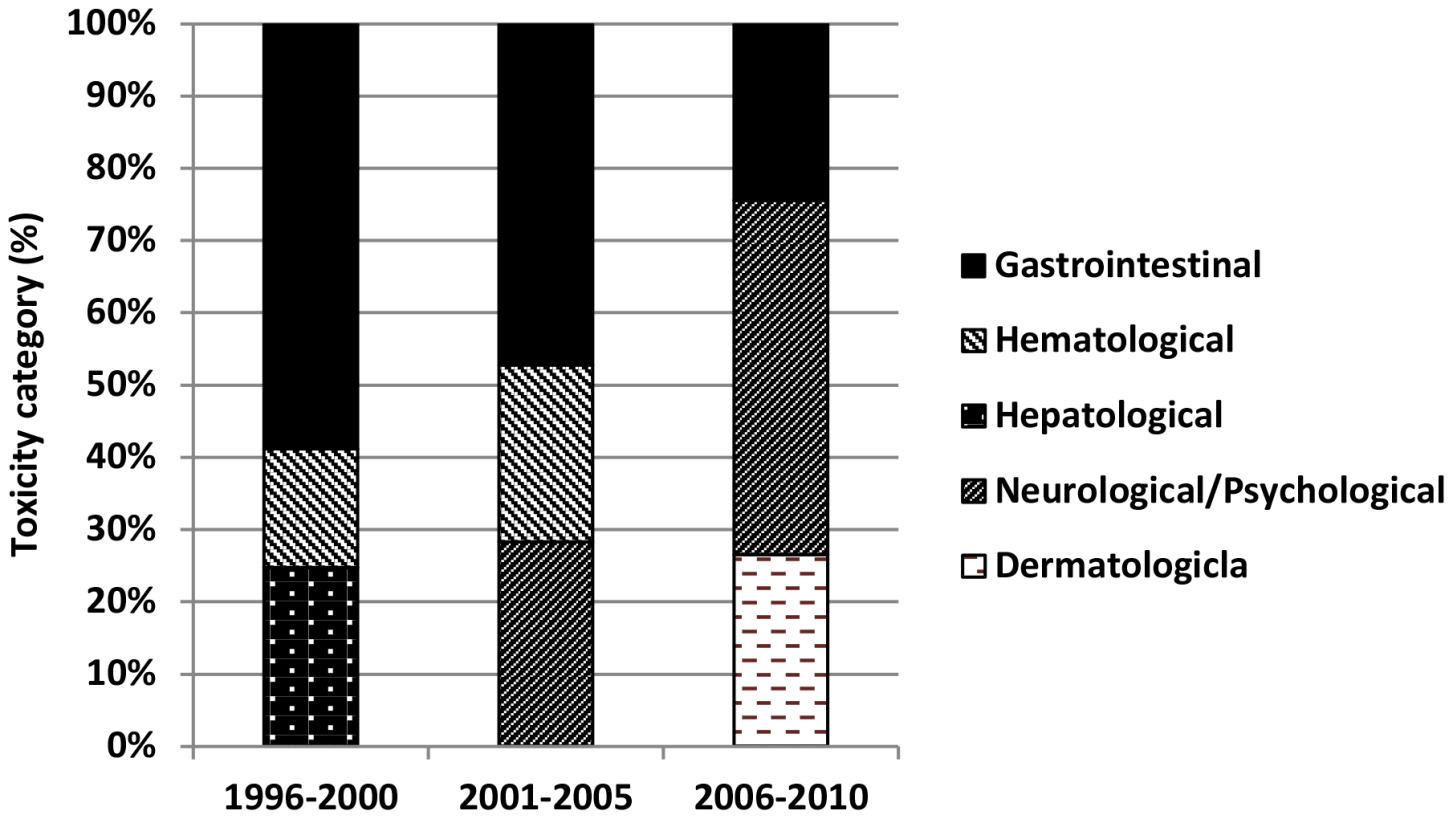
Three toxicity categories that are the cause of most regimen switches per time period.

Among patients who started treatment between 1996 and 2000, the three main adverse events leading to a switch in regimen were gastrointestinal- (36.2% of all toxicity-related stops), hepatological- (15.2%), and hematological-related (10.1%) (Figure 5). In 2001 to 2005 the side effectsmost commonly resulting in switching were gastrointestinal (24.9%), neurological/psychological (14.9%), and hematological (12.9%) toxicity. Finally, between 2006 and 2010 patients most commonly switched regimen due to neurological/psychological (30.7%), dermatological (16.6%), and gastrointestinal toxicity (15.3%).

## Discussion

Between 1996 and 2010, first-line regimens changed from 3TC/AZT-based or d4T/3TC-based regimens with PIs to TDF/3TC or TDF/FTC with NNRTIs. There was no significant reduction in short-term mortality between 1996 and 2010 when controlling for clinical and demographic factors, such as age and CD4 at start of cART, likely because the greatest impact on short-term mortality occurred before this, as a consequence of the introduction of combination therapy [16]. However, there were improvements in CD4 recovery and VL suppression and overall, the short-term incidence of switching decreased significantly since 1996, suggesting that the duration patients spend on a cART-line before switching has improved considerably over time. The incidence of switching to simpler or newer medication in the absence of virological failure or toxicity has increased between 1996 and 2010. As far as we can tell these changes in outcomes are related to new drugs rather than improvements in other aspects of clinical care. This suggests that the changing pattern of first-line cART use over time in the Netherlands, which closely follows changes in guidelines, has done so to the benefit of patients in care.

The pattern of changes in first-line cART regimens in the Netherlands between 1996 and 2010 parallels changes in Dutch national treatment guidelines and the publication of a number of clinical studies [15], [17]. A study by the Swiss HIV cohort study showed that adherence to the national recommendation on cART regimens was associated with better treatment outcomes between 1998 and 2007 [18]. International collaboration cohorts in developed countries have looked at time trends of certain clinical outcomes. The ART cohort collaboration found that while more patients achieved VL suppression to below 500 copies/ml by 6 months that did not result in a reduction in one-year mortality between 1995 and 2003. The Swiss HIV cohort study found that while the one-year incidence of switching did not improve between 2000 and 2005, the proportion of patients that were virological suppressed did improve and CD4 cell count after start of cART showed greater increases over calendar time. Mocroft and colleagues also found no significant change in mortality rates between the early and late cART era [19]. As reported in other countries, toxicity remains the main reason for switching [20]–[23], although newer regimens are associated with improved tolerability [4], [11], [24]. The changes in common side effects resulting in switching coincide with changes in prescribed regimen types between 1996 and 2010 [1]. Studies into the different clinical outcomes have identified similar risk factors. Studies into mortality also reported age, injecting drug use, smoking and AIDS-defining events as risk factors for mortality [16], [25], [26]. Studies into toxicity found the risk to be increased in women, older patients, and amongst patients on older regimens, in accordance with previous work [3]–[7], [23], [27]–[34]. Drug exposure is thought to be influenced by gender-related pharmacokinetics, while polypharmacy in older patients has been shown to significantly increased the chance of serious drug-drug interactions [23], [27]–[30]. As with other studies, our results showed that baseline CD4 count and VL can be a predictor for virological failure, CD4 recovery and VL suppression [35]–[37]. There is no clear explanation why some of the other risk factors identified affect the risk of certain clinical outcomes. For example, our results suggested that MSM have higher risk of toxicity-driven switch compared to heterosexual patients, in accordance with work by Prosperi and colleagues [38]. This may be due to different perceptions of side effects [39].

To our knowledge this is the first attempt to document progress in population-level effectiveness of HIV-treatment in the Netherlands since 1996 across a large number of clinical and non-clinical markers. By using a unique, non-selective dataset to review the changing patterns of cART use coupled with trends in patients' short-term clinical outcomes, it provides a valuable insight into how HIV treatment has changed and the impact this has had on treatment success. The analysis is limited factors typical of cohort data. Comparison of rates of VL suppression is limited by our use of the cut-off of 1,000 copies/ml in the definition of VL suppression. However, we used it in order to compare trends in VL suppression from 1996 to 2010. Analysis of CD4 recovery and VL suppression is restricted by the varying monitoring intervals amongst patients in clinical care. However, as these intervals have lengthened since 1996 and most patients will have a CD4 count and VL test by 12 months; this should not significantly affect the results. The analysis of mortality is limited by the cohort effect, and the fact that the risk of mortality may also depend on a number of factors, such as lifestyle, general health, and co-morbidities, which are not all routinely collected in the dataset. In the analysis we could not control for adherence as adherence data is not routinely collected in ATHENA. Adherence may have improved over time as drugs have become more tolerable [24] and regimens simpler [40], [41]. This and the grouping of regimens into regimen types, makes it inappropriate to associate specific clinical outcomes, such as mortality, to specific regimen types. Consequently, associations observed, such as the increased mortality hazard in patients on TDF/3TC or TDF/FTC with boosted-PIs compared to NNRTI, should be interpreted with caution. The use of marginal structural models may be better suited to carry out this kind of evaluation [42]. The short follow-up available for patients, who started treatment in later years, means that the long-term effect of cART regimen on mortality and toxicity could not be evaluated. The evaluation of the effect of long-term cART use on long-term toxicity and mortality will be important questions to address in the future to ensure the continued high quality standard of care.

The use of first-line cART in the Netherlands closely follows changes in guidelines, to the benefit of patients. While there was no significant improvement in mortality, newer drugs with better tolerability and simpler dosing resulted in improved immunological and virological recovery and reduced incidences of switching due to toxicity and virological failure.

## Supporting Information

Table S1 Adjusted hazard ratio (95% confidence intervals) of 3-year mortality.
(DOCX)

Table S2 Adjusted hazard ratio (95% Confidence intervals) of reaching CD4 increase of 150 cells/mm3 by 12 months.
(DOCX)

Table S3 Adjusted hazard ratio (95% confidence intervals) of virological suppression below 1,000 copies/ml by 12 months.
(DOCX)
