# Supplementary material for: Changes in First-Line cART Regimens and Short-Term Clinical Outcome between 1996 and 2010 in The Netherlands
Source: PLoS One. 2013 Sep 30;8(9):e76071. doi: 10.1371/journal.pone.0076071 (PMC3786897; doi:10.1371/journal.pone.0076071)
Supplement: Table S1 — Adjusted hazard ratio (95% confidence intervals) of 3-year mortality. (DOCX) [file pone.0076071.s001.docx]

Table S1. Adjusted hazard ratio (95% confidence intervals) of 3-year mortality.

| **Variables** | **Model 1: Calendar Time** | | **Model 2: Calendar Time and Regimen Type** | |
| --- | --- | --- | --- | --- |
|  | **Hazard Ratio (95% CI)** | **P-value** | **Hazard Ratio (95% CI)** | **P-value** |
| **Calendar period** |  |  |  |  |
| 1996-2000 | 0.88 (0.63-1.21) | 0.42 | 0.84 (0.54-1.31) | 0.44 |
| 2001-2005 | 1.25 (1.00-1.57) | 0.051 | 1.22 (0.92-1.61) | 0.16 |
| 2006-2010 | [Reference] |  | [Reference] |  |
| **Demographic** |  |  |  |  |
| **Age** |  |  |  |  |
| 5-year increased from 18 years old | 1.27 (1.21-1.33) | <.0001 | 1.27 (1.21-1.33) | <.0001 |
| **Sex** |  |  |  |  |
| Male | [Reference] |  | [Reference] |  |
| Female | 0.85 (0.61-1.18) | 0.33 | 0.85 (0.61-1.18) | 0.32 |
| **Region of Origin** |  |  |  |  |
| Netherlands | [Reference] |  | [Reference] |  |
| European | 0.75 (0.49-1.15) | 0.19 | 0.74 (0.48-1.13) | 0.16 |
| Sub-Saharan Africa | 0.62 (0.41-0.92) | 0.02 | 0.62 (0.41-0.92) | 0.02 |
| Other | 0.77 (0.57-1.04) | 0.08 | 0.77 (0.57-1.04) | 0.08 |
| **Route of transmission** |  |  |  |  |
| Heterosexual | 1.14 (0.86-1.52) | 0.36 | 1.14 (0.86-1.52) | 0.36 |
| MSM | [Reference] |  | [Reference] |  |
| Injecting Drug Use | 2.96 (1.85-4.74) | <.0001 | 2.90 (1.81-4.64) | <.0001 |
| Other | 1.46 (1.05-2.02) | 0.02 | 1.44 (1.04-2.00) | 0.0281 |
| **Smoking** |  |  |  |  |
| Never Smoked | [Reference] |  | [Reference} |  |
| Ever smoke | 1.69 (1.11-2.59) | 0.02 | 1.70 (1.11-2.60) | 0.01 |
| Unknown | 3.57 (2.37-5.39) | <.0001 | 3.57 (2.37-5.39) | <.0001 |
| **Clinical** |  |  |  |  |
| **CD4 cell count at start of cART** |  |  |  |  |
| CD4 <200 | 1.07 (0.67-1.18) | 0.77 | 1.06 (0.67-1.70) | 0.80 |
| CD4 201-350 | 0.86 (0.53-1.41) | 0.55 | 0.87 (0.53-1.43) | 0.58 |
| CD4 351-500 | [Reference] |  | [Reference] |  |
| CD4 >501 | 0.50 (0.19-1.32) | 0.16 | 0.48 (0.18-1.29) | 0.15 |
| **RNA at start of cART** |  |  |  |  |
| RNA <100 000 | [Reference] |  | [Reference] |  |
| RNA 100 000-1 000 000 | 0.91 (0.71-1.18) | 0.49 | 0.92 (0.71-1.19) | 0.51 |
| RNA >1 000 000 | 0.89 (0.56-1.40) | 0.60 | 0.85 (0.54-1.35) | 0.49 |
| **Cummulitive AIDS-defining event** |  |  |  |  |
| No events | [Reference] |  | [Reference] |  |
| 1 event | 3.59 (2.72-4.75) | <.0001 | 3.53 (2.67-4.67) | <.0001 |
| 2 events | 6.19 (4.53-8.46) | <.0001 | 6.05 (4.42-8.29) | <.0001 |
| 3 events | 10.40 (7.04-15.37) | <.0001 | 10.14 (6.85-15.02) | <.0001 |
| 4 events | 8.95 (5.19-15.46) | <.0001 | 8.78 (5.07-15.18) | <.0001 |
| 5 events | 24.81 (11.81-52.12) | <.0001 | 24.88 (11.83-52.32) | <.0001 |
| 6 events | 22.32 (6.92-71.99) | <.0001 | 21.15 (6.54-68.41) | <.0001 |
| 7 events | 49.03 (11.84-203.07) | <.0001 | 50.73 (12.20-210.95) | <.0001 |
| **cART Type** |  |  |  |  |
| 3TC/d4T + PI |  |  | 1.41 (0.72-2.76) | 0.32 |
| 3TC/d4T + Boosted-PI |  |  | 0.94 (0.46-1.92) | 0.86 |
| 3TC/d4T + NNRTI |  |  | 1.49 (0.72-3.11) | 0.28 |
| 3TC/AZT+ PI |  |  | 1.18 (0.70-1.98) | 0.54 |
| 3TC/AZT + Boosted-PI |  |  | 1.16 (0.80-1.68) | 0.43 |
| 3TC/AZT + NNRTI |  |  | 1.26 (0.84-1.89) | 0.27 |
| TDF/FTC or TDF/3TC + Boosted-PI |  |  | 1.49 (1.03-2.15) | 0.04 |
| TDF/FTC or TDF/3TC + NNRTI |  |  | [Reference] |  |
