# Supplementary material for: Changes in First-Line cART Regimens and Short-Term Clinical Outcome between 1996 and 2010 in The Netherlands
Source: PLoS One. 2013 Sep 30;8(9):e76071. doi: 10.1371/journal.pone.0076071 (PMC3786897; doi:10.1371/journal.pone.0076071)
Supplement: Table S2 — Adjusted hazard ratio (95% Confidence intervals) of reaching CD4 increase of 150 cells/mm3 by 12 months. (DOCX) [file pone.0076071.s002.docx]

Table S2. Adjusted hazard ratio (95% Confidence intervals) of reaching CD4 increase of 150 cells/mm^3^ by 12 months.

| **Variables** | **Model 1: Calendar time** | | **Model 2: Calendar time and regimen type** | |
| --- | --- | --- | --- | --- |
|  | **Hazard Ratio (95% CI)** | **P-value** | **Hazard Ratio (95% CI)** | **P-value** |
| **Calendar period** |  |  |  |  |
| 1996-2000 | 0.96 (0.90-1.03) | 0.28 | 1.01 (0.89-1.15) | 0.83 |
| 2001-2005 | 0.94 (0.88-0.99) | 0.03 | 0.97 (0.89-1.06) | 0.46 |
| 2006-2010 | [Reference] |  | [Reference] |  |
| **Demographic** |  |  |  |  |
| **Age** |  |  |  |  |
| 5-year increased from 18 years old | 0.97 (0.96-0.98) | <.0001 | 0.97(0.96-0.99) | 0.0001 |
| **Sex** |  |  |  |  |
| Male | [Reference] |  | [Reference] |  |
| Female | 1.18 (1.08-1.29) | 0.0002 | 1.26 (1.14-1.38) | <.0001 |
| **Region of Origin** |  |  |  |  |
| Netherlands | [Reference] |  | [Reference] |  |
| European | 0.98 (0.89-1.08) | 0.72 | 1.00 (0.90-1.11) | 0.98 |
| Sub-Saharan Africa | 0.72 (0.66-0.80) | <.0001 | 0.71 (0.64-0.78) | <.0001 |
| Other | 0.86 (0.80-0.92) | <.0001 | 0.87 (0.81-0.94) | 0.0007 |
| **Route of transmission** |  |  |  |  |
| Heterosexual | 0.82 (0.76-0.89) | <.0001 | 0.80 (0.74-0.87) | <.0001 |
| MSM | [Reference] |  | [Reference] |  |
| Injecting Drug Use | 0.49 (0.38-0.63) | <.0001 | 0.51 (0.39-0.67) | <.0001 |
| Other | 0.83 (0.74-0.93) | 0.0009 | 0.85 (0.75-0.96) | 0.007 |
| **Clinical** |  |  |  |  |
| **CD4 cell count at start of cART** |  |  |  |  |
| CD4 <200 | 0.83 (0.77-0.91) | <.0001 | 0.84 (0.77-0.92) | 0.0003 |
| CD4 201-350 | 1.00 (0.92-1.09) | 0.99 | 1.02 (0.93-1.12) | 0.66 |
| CD4 351-500 | [Reference] |  | [Reference] |  |
| CD4 >501 | 0.81 (0.72-0.93) | 0.002 | 0.76 (0.65-0.89) | 0.0004 |
| **RNA at start of cART** |  |  |  |  |
| RNA <100 000 | [Reference] |  | [Reference] |  |
| RNA 100 000-1 000 000 | 1.40 (1.33-1.49) | <.0001 | 1.37 (1.29-1.46) | <.0001 |
| RNA >1 000 000 | 2.07 (1.84-2.31) | <.0001 | 1.97 (1.73-2.25) | <.0001 |
| **cART Type** |  |  |  |  |
| 3TC/d4T + PI |  |  | 1.03 (0.85-1.26) | 0.75 |
| 3TC/d4T + Boosted-PI |  |  | 1.30 (1.08-1.57) | 0.01 |
| 3TC/d4T + NNRTI |  |  | 0.74 (0.53-1.02) | 0.06 |
| 3TC/AZT + PI |  |  | 0.88 (0.76-1.03) | 0.12 |
| 3TC/AZT + Boosted-PI |  |  | 1.04 (0.94-1.16) | 0.42 |
| 3TC/AZT + NNRTI |  |  | 0.91 (0.81-1.01) | 0.08 |
| TDF/3TC or TDF/FTC + Boosted-PI |  |  | 1.18 (1.07-1.29) | 0.0006 |
| TDF/3TC or TDF/FTC + NNRTI |  |  | [Reference] |  |
