# Supplementary material for: Changes in First-Line cART Regimens and Short-Term Clinical Outcome between 1996 and 2010 in The Netherlands
Source: PLoS One. 2013 Sep 30;8(9):e76071. doi: 10.1371/journal.pone.0076071 (PMC3786897; doi:10.1371/journal.pone.0076071)
Supplement: Table S3 — Adjusted hazard ratio (95% confidence intervals) of virological suppression below 1,000 copies/ml by 12 months. (DOCX) [file pone.0076071.s003.docx]

Table S3. Adjusted hazard ratio (95% confidence intervals) of virological suppression below 1,000 copies/ml by 12 months.

| **Variables** | **Model 1: Calendar time** | | **Model 2: Calendar time and regimen type** | |
| --- | --- | --- | --- | --- |
|  | **Hazard Ratio (95% CI)** | **P-value** | **Hazard Ratio (95% CI)** | **P-value** |
| **Calendar period** |  |  |  |  |
| 1996-2000 | 0.79 (0.75-0.84) | <.0001 | 0.91 (0.82-1.01) | 0.07 |
| 2001-2005 | 0.90 (0.86-0.95) | <.0001 | 0.95 (0.88-1.01) | 0.12 |
| 2006-2010 | [Reference] |  | [Reference] |  |
| **Demographic** |  |  |  |  |
| **Age** |  |  |  |  |
| 5-year increased from 18 years old | 1.00 (0.99-1.02) | 0.43 | 1.00 (0.99-1.02) | 0.61 |
| **Sex** |  |  |  |  |
| Male | [Reference] |  | [Reference] |  |
| Female | 1.04 (0.96-1.11) | 0.34 | 1.06 (0.98-1.14) | 0.16 |
| **Region of Origin** |  |  |  |  |
| Netherlands | [Reference] |  | [Reference] |  |
| European | 0.92 (0.85-1.00) | 0.06 | 0.90 (0.82-0.98) | 0.02 |
| Sub-Saharan Africa | 0.96 (0.89-1.04) | 0.33 | 0.95 (0.87-1.03) | 0.19 |
| Other | 1.01 (0.95-1.08) | 0.68 | 1.01 (0.94-1.07) | 0.88 |
| **Route of transmission** |  |  |  |  |
| Heterosexual | 0.90 (0.84-0.96) | 0.001 | 0.88 (0.82-0.95) | 0.0004 |
| MSM | [Reference] |  | [Reference] |  |
| Injecting Drug Use | 0.80 (0.68-0.96) | 0.01 | 0.78 (0.64-0.94) | 0.01 |
| Other | 0.92 (0.84-1.01) | 0.07 | 0.92 (0.83-1.02) | 0.11 |
| **Clinical** |  |  |  |  |
| **CD4 cell count at start of cART** |  |  |  |  |
| CD4 <200 | 0.99 (0.92-1.06) | 0.67 | 0.95 (0.88-1.03) | 0.19 |
| CD4 201-350 | 1.02 (0.95-1.09) | 0.62 | 0.96 (0.89-1.04) | 0.34 |
| CD4 351-500 | [Reference] |  | [Reference] |  |
| CD4 >501 | 1.02 (0.91-1.13) | 0.75 | 0.95 (0.84-1.07) | 0.38 |
| **RNA at start of cART** |  |  |  |  |
| RNA <100 000 | [Reference] |  | [Reference] |  |
| RNA 100 000-1 000 000 | 0.65 (0.62-0.68) | <.0001 | 0.67 (0.64-0.71) | <.0001 |
| RNA >1 000 000 | 0.50 (0.45-0.55) | <.0001 | 0.50 (0.45-0.57) | <.0001 |
| **cART Type** |  |  |  |  |
| 3TC/d4T + PI |  |  | 0.87 (0.74-1.03) | 0.11 |
| 3TC/d4T + Boosted-PI |  |  | 0.90 (0.77-1.06) | 0.21 |
| 3TC/d4T + NNRTI |  |  | 0.85 (0.67-1.09) | 0.20 |
| 3TC/AZT + PI |  |  | 0.71 (0.63-0.81) | <.0001 |
| 3TC/AZT + Boosted-PI |  |  | 0.82 (0.75-0.89) | <.0001 |
| 3TC/AZT + NNRTI |  |  | 0.97 (0.88-1.06) | 0.48 |
| TDF/FTC or TDF/3TC + Boosted-PI |  |  | 0.87 (0.81-0.95) | 0.001 |
| TDF/FTC or TDF/3TC + NNRTI |  |  | [Reference] |  |
